# Human papillomavirus infection and atherosclerotic cardiovascular disease: a comprehensive review of epidemiological evidence, molecular mechanisms, and vaccination-mediated protection

**DOI:** 10.3389/fgene.2026.1865685

**Published:** 2026-08-07

**Authors:** Yiming Meng, Jing Sun, Cuicui Kong, Yushu Ma, Tao Yu

**Affiliations:** 1 Department of Central Laboratory, Liaoning Cancer Hospital and Institute, Cancer Hospital of Dalian University of Technology, Shenyang, Liaoning, China; 2 Department of Biobank, Liaoning Cancer Hospital and Institute, Cancer Hospital of Dalian University of Technology, Shenyang, Liaoning, China; 3 Department of Medical Image, Liaoning Jinqiu Hospital, Shenyang, Liaoning, China

**Keywords:** atherosclerosis, cardiovascular disease, epidemiology, human papillomavirus, pathophysiology, vaccination

## Abstract

Emerging epidemiological evidence indicates that persistent infection with high-risk human papillomavirus (HPV) genotypes is independently associated with elevated atherosclerotic cardiovascular disease (CVD) risk, challenging traditional paradigms of cardiovascular risk assessment. This review synthesises current observational data on this association, explores the potential inflammatory, immune, and metabolic mechanisms that may connect HPV to atherogenesis, and evaluates the emerging evidence for cardiovascular protection conferred by HPV vaccination. Large cohort studies report significantly elevated risks of myocardial infarction, stroke, and cardiovascular mortality among women with high-risk HPV infection, independent of conventional risk factors. Mechanistically, HPV oncoproteins E6 and E7 activate nuclear factor-κB (NF-κB)-mediated inflammatory cascades, induce endothelial dysfunction through p53 degradation, and establish a pro-atherogenic systemic inflammatory milieu characterized by elevated interleukin-6 (IL-6) and tumor necrosis factor-α (TNF-α). Preliminary retrospective analyses further indicate that HPV vaccination is linked to a reduced incidence of new-onset cardiovascular and cerebrovascular events, although healthy-user bias and residual confounding limit causal interpretation. Despite these intriguing signals, the bulk of evidence is derived from cross-sectional and retrospective designs, and the direct demonstration of a causal link remains absent. We discuss critical methodological challenges—including confounding by sexual behaviour, socioeconomic status, and co-infections—and outline a roadmap for prospective cohort studies, mechanistic investigations, and rigorous risk-stratification research. Clarifying the nature of the HPV–CVD relationship could open novel avenues for the primary prevention of atherosclerotic disease through vaccination programmes.

## 1 Introduction

### 1.1 Public health context and rationale

Human papillomavirus (HPV) represents the most prevalent sexually transmitted infection globally, with an estimated 291 million women harboring cervical HPV infection at any given time (de Sanjosé et al., 2007). While its causal role in cervical and oropharyngeal malignancies is firmly established, emerging evidence suggests that HPV’s pathogenic impact may extend beyond oncogenesis to encompass cardiovascular pathology (Granja et al., 2024). This gap has spurred the search for non-traditional contributors, including chronic infections. Among these, HPV has emerged as a biologically plausible candidate that may promote atherosclerosis and its clinical sequelae.

Atherosclerosis is now understood as a chronic inflammatory disease (Monaco et al., 2025). Persistent pathogens, including viruses, could sustain a low-grade systemic inflammatory response that accelerates vascular injury. High-risk HPV oncoproteins (e.g., E6 and E7) may influence inflammatory signalling networks and lipid metabolism, thereby indirectly fostering atherogenesis (Granja et al., 2024). Initial epidemiological clues—such as a higher prevalence of HPV among women with coronary artery disease (CAD)—were noted in early integrative reviews (Liang et al., 2023). If the HPV–CVD association proves causal, then HPV prevention strategies, most notably prophylactic vaccination, might yield cardiovascular benefits that extend well beyond their established anti-cancer effects (Reis et al., 2020). Nevertheless, the current evidence base remains inconclusive, and HPV infection should at present be viewed as a risk marker rather than an established independent causal factor.

### 1.2 Aims and structure of this review

**FIGURE 1:**
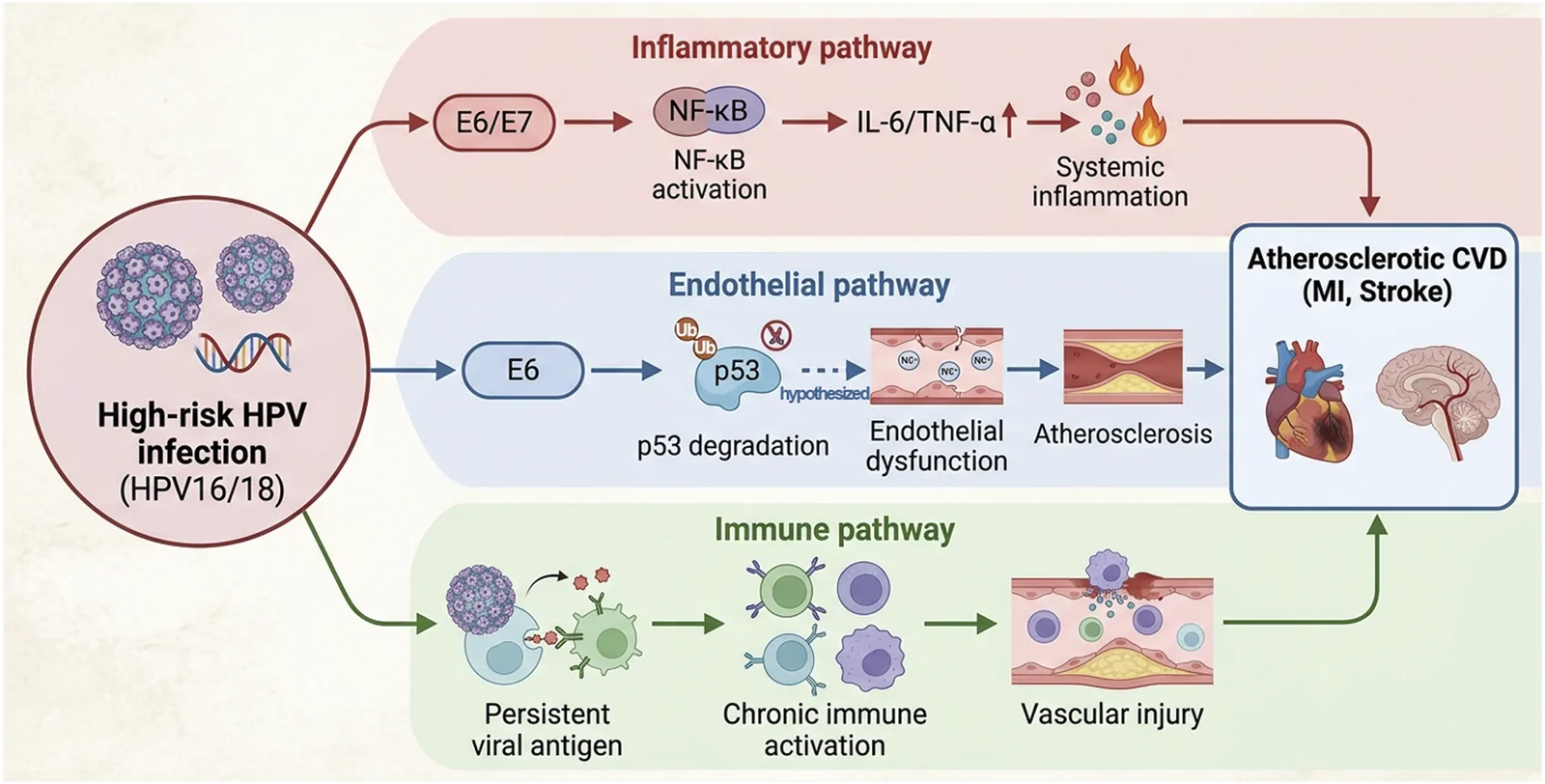
Proposed conceptual framework linking high-risk HPV infection to atherosclerotic cardiovascular disease. Persistent infection with high-risk HPV (HPV-16/18) has been hypothesized to contribute to atherogenesis through three interconnected, putative pathways: (i) an inflammatory pathway, in which the viral oncoproteins E6/E7 may activate NF-κB signaling, leading to elevated IL-6 and TNF-α and systemic inflammation; (ii) an endothelial pathway, in which E6-mediated p53 degradation has been proposed to impair vascular homeostasis and promote endothelial dysfunction and atherosclerosis; and (iii) an immune pathway, in which persistent viral antigen exposure may drive chronic immune activation and vascular injury. These pathways are not mutually exclusive and likely interact. Direct evidence in human vascular tissue remains limited, and the proposed mechanisms require further experimental validation. ASCVD, atherosclerotic cardiovascular disease; HPV, human papillomavirus; IL-6, interleukin-6; MI, myocardial infarction; NF-κB, nuclear factor-kappa B; TNF-α, tumor necrosis factor-alpha; Ub, ubiquitin.

This review aims to critically appraise the evidence linking HPV infection with CVD, delineate areas of consensus and uncertainty, and identify key knowledge gaps. Specific objectives are to: (1) synthesise epidemiological data—from cross-sectional surveys to longitudinal cohort studies—and evaluate the strength and consistency of the observed associations; (2) examine the potential pathophysiological pathways, particularly inflammatory and immune mechanisms, that could connect HPV infection to atherosclerosis; (3) review the nascent evidence on HPV vaccination and cardiovascular outcomes; and (4) discuss methodological challenges that impede causal inference and propose a research agenda for the field (Joo et al., 2019; Saha et al., 2025; Yang et al., 2025). The review focuses on adult populations and on atherosclerotic CVD (especially CAD) and its subclinical markers. Topics confined exclusively to HPV carcinogenesis are discussed only where they intersect with cardiovascular pathology (Figure 1).

## 2 Epidemiological evidence

### 2.1 General population studies: cross-sectional and cohort designs

General population studies provide the foundational evidence for an HPV–CVD link. The most compelling data come from a large prospective cohort study of 163,250 Korean women (median follow-up 8.6 years), which demonstrated that high-risk HPV infection was associated with a significantly elevated risk of atherosclerotic cardiovascular disease mortality (HR = 3.91; 95% CI: 1.85–8.26) after adjusting for age, BMI, smoking status, alcohol intake, physical activity, education level, hypertension, diabetes, lipid-lowering medication use, triglycerides, LDL-C, HDL-C, HOMA-IR, and hsCRP (Joo et al., 2019; Cheong et al., 2024). This study offers robust, population-level support for HPV as a potential non-traditional cardiovascular risk factor.

Other investigations using the United States National Health and Nutrition Examination Survey (NHANES) have reported that HPV positivity is associated with a poorer cardiovascular health score and, in some analyses, with a higher prevalence of self-reported CVD (Li and Song, 2024). However, cross-sectional designs cannot establish temporality, and the possibility of reverse causation—whereby existing vascular disease or its associated immune milieu facilitates HPV persistence—cannot be excluded (Cheong et al., 2024). Prospective cohort studies, which are better suited to inferring directionality, are still sparse, and follow-up periods have generally been too short to capture the full trajectory of atherogenesis Studies included in Table 1 **(** Kuo and Fujise, 2011; Sundaram et al., 2022; Liang et al., 2023; Cheong et al., 2024; Li and Song, 2024 **)** were selected based on three criteria: epidemiological design (cohort, cross-sectional, or case-control), quantitative reporting of HPV–cardiovascular associations with adjusted effect estimates, and adjustment for major cardiovascular confounders; reviews, editorials, and case reports were excluded. The predominance of cross-sectional, female-focused studies in the table reflects the current state of the literature rather than selective inclusion.

**TABLE 1:** The summary of the main epidemiological studies on the association between HPV and CVD.

Study	Population	Sample size	HPV Detection method	Outcome	Adjusted OR/HR (95% CI)	Key confounders adjusted	Ref
Extended surveillance observational cohort study to assess the safety of 9-valent human papillomavirus (9vHPV) vaccine, focusing on rare and serious adverse events	Individuals aged 9–26 years in the United States, enrolled in 6 sites of the Vaccine Safety Datalink (VSD): five Kaiser Permanente organizations (Colorado, Northern California, Northwest, Southern California, Washington) and Marshfield Clinic Health System	1,835,789 doses of 9vHPV vaccine (4 October 2015 – 2 January 2021) 3,814,009 doses of comparator vaccines (Td, Tdap, MenACWY, hepatitis A, varicella; 1 January 2007 – 2 January 2021)	Not applicable	Guillain-Barré syndrome (GBS) Chronic inflammatory demyelinating polyneuropathy (CIDP) Stroke	GBS overall: RR = 0.4 (95% CI: 0.1–0.9) CIDP overall: RR = 2.1 (95% CI: 0.2–28.7) Stroke overall: RR = 0.6 (95% CI: 0.4–1.0)	No formal covariate adjustment was conducted; comparisons were stratified by age (9–17 vs. 18–26 years) and sex (male vs. female)	Sundaram et al. (2022)
A population-based prospective cohort study (Kangbuk Samsung Health Study) examining the association between high-risk human papillomavirus (HR-HPV) infection and cardiovascular disease (CVD) mortality in Korean women	Women aged ≥30 years Underwent comprehensive health screening (including HR-HPV testing) at Kangbuk Samsung Hospital (Seoul/Suwon, South Korea) Free of pre-existing CVD, hysterectomy, and malignancy at baseline Low baseline CVD mortality risk (young/middle-aged, generally healthy)	Initial screened: 178,854 women After exclusions: 163,250 women (final analytic sample) Follow-up person-years: 1,380,953 Median follow-up: 8.6 years (up to 17 years) HR-HPV prevalence: 9.2%	2004–April 2016: Hybrid Capture 2 assay (Qiagen) – detects 13 HR-HPV genotypes April 2016–2018: Roche Cobas HPV assay (real-time PCR) – detects 14 HR-HPV genotypes Results reported as binary (HR-HPV positive vs. negative)	CVD mortality (circulatory system diseases, ICD-10 I00–I99)	ASCVD mortality: 3.91 (1.85–8.26)	Age (timescale) Center (Seoul/Suwon), screening year BMI, smoking status, alcohol intake, regular exercise Education level Hypertension, diabetes, lipid-lowering medication use Triglycerides, LDL-C, HDL-C HOMA-IR (insulin resistance) hsCRP (inflammation marker)	Cheong et al. (2024)
Association between cardiovascular health (CVH) measured by Life’s Essential 8 (LE8) score and human papillomavirus (HPV) infection among U.S. females; cross-sectional analysis using NHANES 2005–2016 data	U.S. females aged 20–59 years	8,264 women	HPV infection: Genotyping of DNA from vaginal swabs using Roche Linear Array HPV Genotyping test High-risk HPV (HR-HPV) infection: Hybrid Capture 2 technology or Cobas test	Prevalence of any HPV infection Prevalence of high-risk HPV (HR-HPV) infection	High CVH level vs. low CVH level HPV infection: 0.67 (0.55–0.82) HR-HPV infection: 0.77 (0.60–0.98)	Age, race, BMI, marital status, education level, family income-to-poverty ratio (PIR), smoking history, age at first sexual intercourse, health insurance coverage, history of oral contraceptive use	Li and Song, (2024)
Cross-sectional study using data from the US National Health and Nutrition Examination Survey (NHANES) 2003–2016	Women aged 20–59 years in the United States	14,371	Self-collected vaginal swab specimens HPV genotyping based on L1 consensus PCR with biotinylated PGMY09/11 primer sets Detects 37 HPV genotypes; classifies 13 types as high-risk and the rest as low-risk	Cardiovascular diseases (CVD), defined as self-reported Coronary heart disease Heart attack Angina pectoris Stroke	HPV infection vs. CVD (fully adjusted): 1.54 (1.15–2.08)	Sociodemographic: age, race/ethnicity, education level, family income-to-poverty ratio, BMI Lifestyle: smoking status, alcohol intake, number of lifetime sexual partners, physical activity	Liang et al. (2023)
Human Papillomavirus and Cardiovascular Disease Among U.S. Women in the National Health and Nutrition Examination Survey, 2003 to 2006	U.S. civilian non-institutionalized women aged 20–59 years	2,450 women	Self-collected vaginal swab specimens; HPV DNA detected by L1 consensus polymerase chain reaction (PCR) followed by type-specific hybridization (Roche Linear Array Assay)	Cardiovascular disease (CVD), defined as self-reported diagnosis of myocardial infarction (heart attack) or stroke	HPV DNA positive vs. negative: 2.30 (1.27–4.16)	Cardiovascular risk factors and management: hypertension, diabetes, waist circumference, triglycerides, HDL-C, log-CRP, log-urinary albumin/creatinine ratio, antihypertensive medications, cholesterol-lowering medications	Kuo and Fujise, (2011)

### 2.2 Associations in high-risk subgroups: menopausal women

Menopausal women represent a particularly informative group because of their sharp increase in cardiovascular risk and persistent background prevalence of HPV. Evidence in this subgroup strongly indicates a relationship between high-risk HPV and CAD. A notable Brazilian cross-sectional study reported that, after adjustment for age, race, traditional cardiovascular risk factors, sexual behaviour, and comorbidities, climacteric women with cervical high-risk HPV DNA had nearly five-fold higher odds of CAD compared with HPV-negative women (Brito et al., 2019). Importantly, no association was observed for low-risk HPV types, suggesting that mechanisms unique to high-risk genotypes—possibly mediated by E6 and E7—are involved in atherosclerosis promotion rather than a generic inflammatory response (Saha et al., 2025). This finding conceptually links the oncogenic properties of HPV to its cardiovascular correlates.

### 2.3 HPV infection and subclinical markers of atherosclerosis

Examining subclinical markers, such as coronary artery calcium (CAC) score and carotid intima-media thickness, offers the opportunity to detect early vascular effects. Direct data on HPV and CAC are scarce. One study, conducted in patients with oropharyngeal cancer, paradoxically found more severe CAC in HPV-negative than HPV-positive individuals (Hytting et al., 2026). Although this observation derives from a highly specialised oncological population—in whom HPV-positive disease is associated with distinct immune profiles, younger age at diagnosis, and better overall prognosis compared with HPV-negative disease—and runs counter to the prevailing hypothesis, it underscores the complexity of the HPV–vascular interface and cautions against extrapolating findings from cancer populations to the general population. Critically, this paradoxical finding may be substantially or even entirely attributable to the well-documented disparity in tobacco exposure between HPV-positive and HPV-negative oropharyngeal cancer patients, rather than reflecting any genuine protective vascular effect of HPV infection itself. HPV-negative oropharyngeal cancers are aetiologically driven predominantly by heavy and prolonged tobacco and alcohol use, whereas HPV-positive oropharyngeal cancers disproportionately affect younger, non-smoking individuals whose disease arises through sexual transmission of high-risk genotypes (Gillison et al., 2008). Given that cumulative tobacco exposure is one of the most potent accelerators of coronary artery calcification-operating through sustained endothelial oxidative stress, vascular smooth muscle cell calcification, and chronic systemic inflammation-the observed between-group difference in CAC severity may simply recapitulate the between-group difference in pack-year smoking history (Csordas and Bernhard, 2013). If smoking history was not rigorously quantified and adjusted for as a continuous variable in this study, the paradoxical CAC finding cannot be interpreted as evidence against the HPV–CVD hypothesis; rather, it should be understood as a reflection of the profound cardiovascular toxicity of tobacco in the HPV-negative oropharyngeal cancer demographic (Araujo et al., 2025). This interpretation further reinforces the argCritically, this paradoxical ument, elaborated in Section 5.2, that tobacco smoking represents an indispensable confounder that must be measured with biochemical precision and adjusted for with methodological rigour in all future HPV–cardiovascular investigations.

Epidemiological data on HPV and carotid intima-media thickness, arterial stiffness, or other vascular functional measures are largely absent. No firm conclusion can yet be drawn regarding the association between HPV and any specific subclinical atherosclerosis marker; this remains an important knowledge gap (Hytting et al., 2026).

## 3 Potential pathophysiological mechanisms linking HPV to atherosclerosis

### 3.1 Systemic inflammation and immune activation

Chronic low-grade systemic inflammation represents a cardinal pathophysiological mechanism underlying atherosclerotic plaque initiation and progression (Kong et al., 2022). Persistent high-risk HPV infection functions as a continuous antigenic stimulus, engaging pattern recognition receptors (PRRs) such as Toll-like receptors (TLRs) on innate immune cells and subsequently activating adaptive immune responses through antigen presentation pathways. At the molecular level, HPV oncoproteins E6 and E7 directly interact with host signaling cascades: E7 binds to and inactivates retinoblastoma protein (Rb), leading to E2F-mediated transcriptional activation of NF-κB target genes, while E6-mediated p53 degradation removes a critical brake on inflammatory gene expression. This dual mechanism results in sustained elevation of pro-inflammatory cytokines including TNF-α, IL-6, interleukin-1β (IL-1β), and monocyte chemoattractant protein-1 (MCP-1) within the infected mucosal epithelium (Saha et al., 2025). However, given that high-risk HPV infection is fundamentally restricted to the mucosal epithelium, a critical mechanistic question arises: how do locally produced pro-inflammatory mediators successfully reach the systemic circulation in quantities sufficient to drive atherogenesis at distant vascular sites? Several non-mutually exclusive mechanisms may account for this translocation. First, HPV-infected keratinocytes have been demonstrated to release extracellular vesicles-including exosomes of approximately 30–150 nm in diameter-that carry viral oncoproteins, microRNAs, and pro-inflammatory cargo capable of traversing mucosal barriers and entering the bloodstream, where they may activate circulating monocytes and vascular endothelial cells at sites remote from the primary infection (Cakir et al., 2025). Second, locally activated mucosal macrophages and dendritic cells, reprogrammed toward a pro-inflammatory M1 phenotype by E6/E7-mediated NF-κB activation, may undergo systemic migration via draining lymphatics and the thoracic duct, disseminating inflammatory signals to the arterial wall (Gholami et al., 2026). Third, chronically elevated mucosal IL-6 may transactivate hepatic acute-phase responses through the JAK-STAT3 signalling axis, generating systemic elevations of C-reactive protein and fibrinogen that independently promote endothelial dysfunction. Collectively, these pathways provide a plausible biological bridge through which a compartmentalised mucosal infection may export sufficient pro-inflammatory signalling to foster a systemic pro-atherogenic environment.

Observational studies provide circumstantial support. The strong link between high-risk HPV and CAD in menopausal women has been attributed, in part, to persistent immune-inflammatory activation (Granja et al., 2024). At the molecular level, E6-mediated degradation of p53 has been hypothesised to alter macrophage function, reducing apoptosis and favouring a pro-inflammatory phenotype that exacerbates lipid deposition and vascular wall inflammation (Palatnic et al., 2024). This provides a plausible molecular bridge between viral infection and systemic inflammation.

### 3.2 Impact on metabolic syndrome components

Metabolic syndrome and its constituents are well-established cardiovascular risk factors. Theoretically, HPV-driven chronic inflammation could disturb metabolic homeostasis—by impairing adipose tissue function or promoting insulin resistance—and thereby indirectly increase atherosclerotic risk (Huang et al., 2016). A potential direct intracellular pathway has also been proposed, whereby HPV oncoproteins E6 and E7 disrupt PPAR-γ signaling and lipid droplet biogenesis independently of systemic inflammation, though this mechanism remains insufficiently characterized. However, cross-sectional studies examining HPV associations with obesity, insulin resistance, and dyslipidaemia have yielded inconsistent results, partly reflecting confounding by obesity, sexual behaviour, and socioeconomic position, and partly the divergent age distributions of HPV prevalence and metabolic syndrome burden (Chen et al., 2025).

These inconsistencies partly reflect inadequate adjustment for shared confounders-particularly obesity, sexual behaviour patterns, and socioeconomic position-which simultaneously influence both HPV acquisition and metabolic health (Huang et al., 2016). Inadequate adjustment can easily generate spurious associations. Furthermore, the population distribution of HPV and metabolic risk differs markedly by age: HPV prevalence is high among young women who have a low burden of metabolic syndrome, whereas the reverse is true for menopausal women (Smith et al., 2008; Chan et al., 2009). Taken together, current cross-sectional evidence does not convincingly demonstrate an independent effect of HPV on metabolic syndrome components; at most, weak subgroup-specific associations may exist and require prospective validation.

### 3.3 Direct versus indirect effects on vascular endothelial cells

Endothelial dysfunction is an initiating step in atherosclerosis (Libby and Soehnlein, 2025). Two broad hypotheses exist regarding HPV’s role. The “direct” hypothesis posits that HPV particles or nucleic acids may infect vascular endothelial cells and disrupt cell-cycle control or provoke oxidative stress (Tonhajzerova et al., 2019). Although isolated reports have claimed detection of HPV DNA in atherosclerotic plaques, these findings are based on small samples, suffer from potential contamination, and have not been reliably replicated.

The “indirect” hypothesis, which currently enjoys stronger support, proposes that HPV acts principally through systemic inflammation and immune dysregulation that secondarily impair endothelial function (Reis et al., 2020). As discussed in Section 2.3, the paradoxical CAC finding in HPV-negative oropharyngeal cancer patients is most plausibly attributable to differential tobacco exposure rather than a genuine protective vascular effect of HPV, further supporting the indirect rather than direct vascular pathway (Hytting et al., 2026; Nishikawa et al., 2026). Definitive evidence of productive HPV infection in vascular endothelial cells is still lacking, and the indirect pathway remains the more parsimonious explanation (Chan et al., 2024). To definitively resolve this question, future investigators should employ modern high-resolution molecular techniques capable of detecting low-abundance viral nucleic acids with precise spatial localisation within complex tissue architectures. Specifically, spatial transcriptomics-which simultaneously maps genome-wide gene expression profiles at single-cell resolution across intact tissue sections-would enable researchers to determine whether HPV transcriptional activity co-localises with endothelial cell markers within atherosclerotic plaques, thereby distinguishing true endothelial infection from incidental viral DNA contamination or passive uptake. Complementarily, highly sensitive multiplex *in situ* hybridization (MISH), such as RNAscope-based assays, permits simultaneous visualisation of HPV E6/E7 mRNA transcripts alongside cell-type-specific markers within formalin-fixed paraffin-embedded plaque specimens, providing definitive evidence of productive viral replication at the single-cell level rather than merely detecting residual viral DNA of uncertain biological significance. The concurrent application of both techniques to well-characterised atherosclerotic plaque biobanks, with rigorous negative controls and HPV genotype-specific probes, would either conclusively establish or rule out direct endothelial tropism of high-risk HPV, fundamentally clarifying whether the direct hypothesis warrants further mechanistic investigation (Suresh et al., 2021; Chan et al., 2024; Lai et al., 2025).

## 4 HPV vaccination and cardiovascular protection: early observations

### 4.1 Observational studies on vaccination and cardiovascular outcomes

Retrospective analyses of large databases provide preliminary hints that HPV vaccination may be associated with lower cardiovascular risk (Vilela et al., 2025; Yang et al., 2025). A meta-analysis of seven studies with nearly 250,000 participants demonstrated that HPV-positive individuals had a 40% higher risk of CVD and double the risk of CAD, leading the authors to propose that “HPV vaccination could be explored as a preventive measure against cardiovascular complications” (Vilela et al., 2025). A separate NHANES-based analysis documented a similar trend (Liang et al., 2023). A recent prospective matched cohort study of approximately 3 million Swedish women—the first European population-level evidence on this topic—found that HPV infection was associated with elevated CVD incidence (HR 1.07) and mortality over up to 17 years of follow-up, providing indirect but powerful support for the hypothesis that preventing HPV infection could lower CVD risk (Deng and Lei, 2025).

Nevertheless, these observational data have important limitations. Retrospective designs cannot fully exclude “healthy-user bias”—the tendency for vaccine recipients to adopt healthier lifestyles and exhibit better healthcare adherence. This bias is particularly pernicious here because vaccination uptake is strongly predicted by socioeconomic privilege, health literacy, and proactive healthcare engagement, precisely the characteristics that independently confer lower cardiovascular risk, rendering conventional multivariable adjustment insufficient (Liang et al., 2023). Future retrospective studies should therefore employ advanced causal inference methods, including: (i) propensity score matching (PSM) or inverse probability of treatment weighting (IPTW) to balance groups across key determinants of vaccination uptake; (ii) instrumental variable (IV) analysis using geographical variation in programme rollout, temporal policy changes, or school-level mandates to isolate exogenous vaccination exposure; (iii) active comparator designs replacing unvaccinated controls with recipients of a comparator vaccine to cancel the healthy-user effect; (iv) negative outcome control analyses to empirically quantify residual bias; and (v) Mendelian randomisation using genetic instruments for immune responsiveness or HPV susceptibility where large biobank cohorts are available (Austin, 2011; Davey Smith and Hemani, 2014).

Follow-up periods in existing studies are relatively short and largely confined to young and middle-aged adults, whereas atherosclerosis develops over decades (Yang et al., 2025). Although associations persist after multivariable adjustment, residual confounding by unmeasured factors remains plausible.

### 4.2 Hypothesised mechanisms of vaccine-mediated cardiovascular protection

Drawing on the presumed pro-atherogenic pathways of HPV infection, several mechanisms have been advanced to explain the putative cardiovascular benefit of vaccination (Dutta et al., 2024). The primary hypothesis is that vaccination prevents initial and persistent high-risk HPV infection, thereby blocking the cascade of chronic inflammation and immune activation that may injure the vascular endothelium (Reis et al., 2020).

Specifically, averting long-term infection could (i) eliminate a source of sustained low-grade systemic inflammation, lessening indirect endothelial damage, and (ii) potentially prevent any residual direct vascular effects of viral components (Tonhajzerova et al., 2019; Liang et al., 2023). The hypothesis that vaccination acts by improving metabolic parameters such as lipid profiles has garnered little support; most observational analyses adjust for metabolic covariates and the vaccine–CVD association persists, implying that any benefit is independent of gross metabolic improvements or is mediated by subtler changes not yet captured (Yang et al., 2025).

It must be stressed that these mechanistic hypotheses are largely extrapolated from the pathophysiology of HPV infection itself (Yang et al., 2025). Direct studies demonstrating that vaccination reduces human CVD risk through these specific pathways are lacking.

## 5 Key challenges, controversies, and barriers to translation

### 5.1 Study design limitations and the challenge of causal inference

The most fundamental barrier is that the existing evidence base is dominated by cross-sectional studies, which cannot establish temporality (Joo et al., 2019). Although associations between HPV and CAD or subclinical markers are repeatedly observed, it remains impossible to distinguish whether infection promotes vascular disease or whether pre-existing vascular pathology and related immune states facilitate HPV persistence (Palatnic et al., 2024). Prospective cohort studies are the critical tool to address temporality, yet they remain few and not entirely concordant Cheong et al., 2024). Even in prospective designs, measurement and control of important confounders—including sexual behaviour, socioeconomic position, and co-infections—have often been insufficient, and residual confounding may distort effect estimates (Chan et al., 2024; Cheong et al., 2024). A cautious summary is therefore that an epidemiological association exists, but its causal direction and the magnitude of any independent effect remain to be established by robust prospective research.

### 5.2 Confounding: behaviour, socioeconomic status, and co-infections

Demonstrating the independence of the HPV–CVD association requires rigorous control of complex confounders (Palatnic et al., 2024). Three categories of confounding are of paramount importance and must be considered jointly rather than sequentially: sexual behaviour, socioeconomic status, and tobacco smoking. Each operates through distinct biological and epidemiological pathways, and each is capable of independently generating a spurious HPV–CVD association if inadequately measured or adjusted for. HPV infection is tightly linked to sexual behaviour, and the number of lifetime sexual partners correlates with smoking, alcohol use, and other lifestyle factors that are themselves classic CVD risk factors (Palatnic et al., 2024). If studies do not accurately measure and adjust for these behavioural covariates, the observed association may reflect a shared behavioural risk background (Liang et al., 2023).

Socioeconomic status is another deep confounder: lower socioeconomic position is associated with elevated HPV risk, reduced healthcare access, and higher CVD burden (Zhao et al., 2023; Cheong et al., 2024). Inadequate adjustment can overestimate HPV-specific effects. Tobacco smoking represents a particularly critical and underappreciated confounder that warrants dedicated consideration. Smoking exerts a profound dual influence on both sides of the HPV–CVD association simultaneously (Lilja-Fischer et al., 2025). With respect to HPV biology, available evidence suggests that tobacco use and nicotine may weaken local cervical immune surveillance, including reductions in Langerhans cells and other local immune responses, thereby potentially impairing spontaneous HPV clearance and favoring the persistence of high-risk HPV infection. Furthermore, nicotine has been reported in vitro to promote the proliferation of HPV-immortalized cervical epithelial cells via the RPS27a-Mdm2-P53 pathway (Chen and Wang, 2019; Utami et al., 2021). Epidemiological evidence consistently demonstrates that current and former smokers exhibit significantly higher rates of HPV persistence and progression to cervical intraepithelial neoplasia compared with never-smokers, independent of sexual behaviour variables. With respect to cardiovascular pathology, tobacco use is simultaneously one of the most potent modifiable risk factors for atherosclerosis, acting through endothelial oxidative stress, systemic upregulation of IL-6 and TNF-α, dyslipidaemia, and platelet hyperactivation-the same inflammatory mediators implicated in HPV-driven atherogenesis (Kobayashi et al., 2024). An important methodological limitation in the current literature is the potential for residual confounding by tobacco exposure. Smoking is itself associated with systemic inflammation and endothelial dysfunction, which may contribute to cardiovascular risk through mechanisms that partially overlap with those hypothesized for HPV, although the two should not be regarded as biologically equivalent. In addition, smoking tends to cluster with other behavioral and sociodemographic determinants of cardiovascular disease. Although existing studies have adjusted for smoking and other conventional risk factors, such adjustment may not adequately capture the full complexity of tobacco exposure, including smoking intensity, cumulative burden, passive exposure, and time since cessation. Therefore, tobacco exposure remains a plausible source of residual confounding in the reported association between HPV and cardiovascular disease. However, current evidence is insufficient to conclude that smoking alone fully accounts for this association (Cheong et al., 2024; Dutta et al., 2024). Future studies should incorporate more detailed quantitative smoking histories, including duration of smoking and pack-year accumulation, and, where possible, use biochemically validated measures of tobacco exposure such as cotinine or other nicotine metabolites rather than relying solely on self-reported smoking data. Larger sample sizes and longer follow-up periods would also help to clarify the association between smoking, high-risk HPV infection, and the development of high-grade cervical intraepithelial lesions (Kayar et al., 2025). Finally, immune competence and co-infections represent key biological confounders. Individual variation in immune function can simultaneously affect HPV clearance and atherosclerotic susceptibility (Tonhajzerova et al., 2019). Moreover, HPV frequently co-occurs with other infections, notably HIV, which independently increases both HPV persistence and cardiovascular risk (Machado de Carvalho et al., 2021; Kawai et al., 2025). Failure to control for HIV-related immunosuppression can erroneously attribute CVD risk to HPV.

### 5.3 Mechanistic depth and the translational gap

Despite suggestive epidemiological data, the pathophysiological pathways connecting HPV to atherosclerosis remain inadequately defined. The most commonly cited mechanism—systemic inflammation—is supported mainly by indirect evidence. Few studies have simultaneously measured HPV status and serial inflammatory biomarker dynamics, and cross-sectional designs cannot resolve whether inflammation is cause or consequence (Lazzari et al., 2025; Yang et al., 2026). Consequently, although inflammation is a plausible biological mediator, its independent quantitative contribution and temporal sequence in the HPV–CVD pathway remain to be rigorously quantified in prospective cohorts with serial inflammatory biomarker profiling and comprehensive confounding control.

The hypothesis of direct vascular infection by HPV is particularly contentious. Claims of HPV DNA in atherosclerotic plaques are based on small samples and face technical challenges, including potential contamination and false positives. The biological plausibility of productive HPV infection in endothelial cells is seriously questioned (Lawson et al., 2015). At present, the more reasonable stance is that direct endothelial infection is unlikely, although definitive large-scale studies are warranted to settle the issue. To achieve conclusive resolution of this question, future investigations should employ a coordinated suite of next-generation sequencing and spatially resolved molecular techniques rather than relying on conventional bulk PCR-based HPV DNA detection, which is inherently vulnerable to contamination artefacts and cannot provide cellular-level resolution. Specifically, we recommend the following methodological approaches. First, single-cell RNA sequencing (scRNA-seq) applied to freshly dissociated atherosclerotic plaque tissue would enable transcriptomic profiling at single-cell resolution, allowing investigators to determine whether endothelial cells, vascular smooth muscle cells, or plaque-resident macrophages harbour HPV transcriptional activity-as opposed to merely containing extracellular or contaminating viral DNA-and to characterise any associated perturbations in host gene expression programmes governing endothelial activation, inflammatory signalling, and cell cycle regulation (Puram et al., 2017; Fernandez et al., 2019; Wirka et al., 2019). Second, spatial transcriptomics platforms, such as 10x Visium or Slide-seq, would preserve the histological architecture of the plaque while simultaneously mapping HPV RNA localisation, thereby distinguishing genuine intracellular infection from luminal or adventitial viral deposition (Stickels et al., 2021; Cheng and Quertermous, 2025). Third, chromatin immunoprecipitation sequencing (ChIP-seq) targeting HPV E6 and E7 binding partners-particularly p53 and pRb respectively-in plaque-derived cell populations would provide functional evidence of oncogenic protein activity, which would constitute far more compelling evidence of biologically meaningful infection than viral DNA presence alone. Fourth, long-read nanopore sequencing or PacBio HiFi sequencing could determine whether episomal or integrated HPV genomes are present within plaque-resident cells, as chromosomal integration of the HPV genome is a hallmark of biologically active, replication-competent infection rather than passive DNA deposition (Wang et al., 2024). Fifth, highly multiplexed spatial proteomics approaches, such as imaging mass cytometry or CODEX, could simultaneously visualize HPV capsid proteins, endothelial markers, and inflammatory mediators within intact plaque sections, providing direct morphological evidence of cellular co-localization (Goltsev et al., 2018). The rigorous application of these complementary technologies-ideally in large, well-characterized biobank cohorts with matched HPV-positive and HPV-negative plaque specimens-would either firmly establish endothelial tropism as a genuine mechanistic pathway or definitively exclude it, thereby substantially clarifying the translational relevance of the direct infection hypothesis.

### 5.4 Clinical implications and current recommendations

Despite the accumulating evidence, it is premature to incorporate HPV screening into cardiovascular risk assessment algorithms (Pradhan et al., 2026). Current cardiovascular risk calculators (e.g., Framingham Risk Score, ASCVD Risk Estimator) do not include infectious parameters, and the incremental predictive value of adding HPV status remains unquantified.

However, the potential cardiovascular co-benefit of HPV vaccination strengthens the public health case for achieving high vaccination coverage, particularly in populations with suboptimal uptake (Yang et al., 2025). Clinicians should be aware that women with documented persistent high-risk HPV infection may represent a group warranting intensified cardiovascular risk factor modification, although this recommendation awaits validation in intervention trials.

From a research ethics perspective, the existing signals justify prioritizing HPV-CVD studies in research funding allocation, but do not yet support withholding HPV vaccination from control groups in cardiovascular outcome trials, given vaccination’s established cancer prevention benefits (Kuo and Fujise, 2011; Palatnic et al., 2024).

## 6 Future directions and translational prospects

### 6.1 Advancing prospective cohort studies and trial designs

To overcome the current impasse in causal inference, large, rigorously designed prospective cohort studies are essential. These should include baseline assessment of HPV status (with genotyping, viral load quantification, and distinction between transient and persistent infection) and long-term follow-up (ideally ≥10 years) to capture incident cardiovascular events. Existing cohorts such as the Korean women’s study and the report on HPV and cardiovascular mortality provide useful starting points but could be strengthened in their control of confounders (Palatnic et al., 2024).

To better evaluate the cardiovascular effects of HPV vaccination, future research should prioritize prospective studies with detailed immunization records, longer follow-up, and more granular clinical endpoint assessment. Long-term surveillance of vaccinated populations will be important to determine whether the observed association with reduced cardiovascular and cerebrovascular risk is sustained over time. Where feasible, future studies should also examine specific outcomes, such as myocardial infarction or stroke, and incorporate biomarker-based or mechanistic investigations to clarify the underlying pathways (Yang et al., 2025). In addition, quasi-experimental approaches using real-world data—such as instrumental-variable analyses—may offer complementary evidence (Carter et al., 2024). The protective signals seen in database-driven studies supply a compelling rationale for such work.

### 6.2 Deepening molecular mechanism and biomarker research

Mechanistic studies must move beyond descriptive associations to delineate how local HPV infection generates systemic and vascular pathologies (Dutta et al., 2024; Lazzari et al., 2025). A priority is to assess the presence, cellular tropism, and transcriptional activity of high-risk HPV within atherosclerotic plaques using rigorous techniques such as multiplex in-situ hybridisation and single-cell sequencing, applied to adequately sized samples with careful distinction between active infection and residual viral DNA (Lawson et al., 2015).

Concurrently, efforts should focus on identifying intermediate biomarkers that capture the specific pathological processes linking HPV to CVD (Lawson et al., 2015; Tonhajzerova et al., 2019). Candidate markers could include host response molecules indicative of E6/E7 activity, and markers of endothelial dysfunction such as soluble intercellular adhesion molecule-1 or endothelial microparticles (Deng and Lei, 2025; Gholami et al., 2026). These must be evaluated in prospective cohorts to determine whether they differ between infected and uninfected individuals and whether they independently predict cardiovascular events.

### 6.3 Risk stratification and personalised prevention: a cautious outlook

Integrating HPV status into cardiovascular risk-stratification algorithms is a forward-looking but premature proposition (Lazzari et al., 2025). Major obstacles include: the absence of established causality; the concentration of current evidence in specific populations (e.g., menopausal women), leaving its generalisability to men, younger individuals, and diverse ethnic groups unknown; imprecision in risk estimation due to inter-study heterogeneity; and a lack of cost-effectiveness data for incorporating HPV testing into primary CVD prevention (Deng and Lei, 2025; Lazzari et al., 2025; Yang et al., 2026).

A stepwise research approach is advisable: first, demonstrate the incremental predictive value of HPV infection—especially persistent high-risk infection—in prospective cohorts (Cheong et al., 2024). Second, evaluate its predictive performance in high-risk subgroups, such as those with concomitant inflammatory conditions or poorly controlled traditional risk factors. Only if a clear and quantifiable net clinical benefit is demonstrated should its potential clinical application in personalised prevention be considered (Yang et al., 2026).

## 7 Conclusion and perspectives

Emerging evidence suggests that high-risk HPV infection may be a potential non-traditional risk factor for atherosclerotic cardiovascular disease. This hypothesis is supported by converging epidemiological observations and mechanistic studies implicating chronic inflammation, endothelial dysfunction, and immune signaling pathways. Nevertheless, the available data are still preliminary, and additional well-designed studies are needed to determine whether this association is causal and whether it has meaningful implications for cardiovascular prevention (Saha et al., 2025). Mechanistic plausibility is suggested by studies showing that HPV16 E6 can alter NF-κB signaling and anti-apoptotic pathways. In addition, review-level evidence proposes that persistent high-risk HPV infection may promote vascular inflammation through E6/E7-related disruption of host regulatory pathways, although direct vascular and endothelial mechanisms remain insufficiently established (James et al., 2006).

The nascent observation that HPV vaccination may confer cardiovascular protection—if confirmed in rigorous prospective studies—would represent a paradigm shift in preventive cardiology, positioning an infectious disease vaccine as a novel tool for atherosclerosis prevention (Cheong et al., 2024; Deng and Lei, 2025). Such a finding would parallel the cardiovascular benefits observed with influenza vaccination and underscore the broader principle that modulating chronic inflammation, regardless of its source, can reduce vascular risk.

Nevertheless, critical knowledge gaps persist. The causal nature of the HPV-CVD association remains unproven; existing data cannot exclude the possibility that HPV is merely a marker of unmeasured confounders such as sexual behavior patterns, socioeconomic disadvantage, or immune senescence (Cheong et al., 2024). The molecular mechanisms require deeper interrogation through prospective biomarker studies and direct examination of HPV presence and activity in atherosclerotic plaques using state-of-the-art techniques (Lippi et al., 2007). The cardiovascular effects of vaccination demand evaluation in randomized trials or robust quasi-experimental designs that minimize healthy-user bias.

Looking forward, we propose a coordinated research agenda encompassing: (i) large-scale prospective cohorts with serial HPV genotyping, viral load quantification, and adjudicated cardiovascular endpoints; (ii) mechanistic studies employing systems biology approaches to map HPV-induced perturbations in inflammatory, metabolic, and vascular networks; (iii) secondary analyses of existing HPV vaccine trials with extended cardiovascular follow-up; and (iv) development and validation of integrated risk prediction models incorporating HPV status alongside traditional risk factors.

If the HPV-CVD link proves causal, the implications extend beyond individual patient care to population health strategy. HPV vaccination programs, already justified by cancer prevention, would gain additional impetus from cardiovascular co-benefits, potentially improving cost-effectiveness and expanding target populations to include older adults and males (Giannone et al., 2022). Conversely, failure to confirm causality would itself be informative, redirecting research toward other infectious or inflammatory contributors to residual cardiovascular risk (Yang et al., 2025).

In conclusion, the intersection of HPV infection and cardiovascular disease represents a frontier in both virology and cardiology, with the potential to reshape our understanding of atherosclerosis etiology and expand the toolkit of primary prevention. Rigorous science, free from premature clinical translation, will determine whether this intriguing association heralds a new chapter in cardiovascular medicine.
